# Comparative evaluation of four immunoassays for *Plasmodium vivax* serological detection using recombinant PvMSP1-42 antigen

**DOI:** 10.1371/journal.pntd.0014570

**Published:** 2026-08-05

**Authors:** Jiahui Xu, Tao Yu, Zihan Zhao, Yaobao Liu, Yinyue Li, Niming Zhang, Tang Xu, Xinlong He, Eun-Taek Han, Jin-Hee Han, Zhiyong Tao, Didier Menard, Feng Lu

**Affiliations:** 1 School of Basic Medical Sciences, Faculty of Medicine, Yangzhou University, Yangzhou, China; 2 University Key Laboratory of Jiangsu Province for Nucleic Acid and Cell Fate Regulation Yangzhou University, Yangzhou, China; 3 Affiliated Hospital of Yangzhou University, Yangzhou University, Yangzhou, China; 4 Shandong Institute of Parasitic Diseases, Shandong First Medical University and Shandong Academy of Medical Sciences, Jining, Shandong, China; 5 National Health Commission Key Laboratory of Parasitic Disease Control and Prevention, Jiangsu Provincial Key Laboratory on Parasite and Vector Control Technology, Jiangsu Provincial Medical Key Laboratory, Jiangsu Institute of Parasitic Diseases, Wuxi, China; 6 Department of Medical Environmental Biology and Tropical Medicine, Kangwon National University School of Medicine, Chuncheon, Korea; 7 Key Laboratory of Infection and Immunity of Anhui Higher Education Institutes, Bengbu Medical University, Bengbu, China; 8 Malaria Genetics and Resistance Team (MEGATEAM), UR 3073 - Pathogens Host Arthropods Vectors Interactions Unit, Université de Strasbourg, Strasbourg, France; 9 Malaria Parasite Biology and Vaccines, INSERM Unit 1347 - ParasitInnov, Institut Pasteur, Université Paris Cité, Paris, France; 10 Laboratory of Parasitology and Medical Mycology, CHU Strasbourg, Strasbourg, France; 11 Institut universitaire de France (IUF), Paris, France; Shanghai Jiao Tong University School of Medicine, CHINA

## Abstract

**Background:**

Serological surveillance is a vital component in the control and elimination of *Plasmodium vivax*, particularly during low-transmission or elimination phases. Immunoassays are core tools for malaria serological surveillance. Among them, protein arrays, dot blot, enzyme-linked immunosorbent assay (ELISA), and Western blot are widely used, each with distinct diagnostic and operational characteristics.

**Methods:**

We evaluated a recombinant His-tagged PvMSP1–42 protein using sera from immunized mice, *P. vivax*-infected patients, and healthy individuals across the platforms under standardized conditions.

**Results:**

All immunoassays demonstrated high reproducibility but varied in sensitivity and sample requirements. Protein arrays enabled a broad detection range (3.13–200 ng/μl) with minimal protein and antibody use, with performance further enhanced using a protein stabilizing diluent. Dot blot and Western blot exhibited similar detection thresholds (6.25–200 ng/μl), although dot blot showed improved sensitivity at low serum dilutions when using Protein Microarray Spot Diluent. ELISA achieved the highest sensitivity, detecting specific antibodies at dilutions up to 1:128,000, but plateaued beyond 50 ng/μl of antigen. Apart from the dot blot, the other methods demonstrated both sensitivity and specificity exceeding 90%, whereas dot blot sensitivity remained lower at 80%.

**Conclusions:**

Protein arrays offered a favorable balance between sensitivity, throughput capability and sample conservation. ELISA was the most effective for detecting low antibody titers. Dot blot and Western blot remained useful, depending on resource availability and experimental goals. These findings provide a practical reference for selecting appropriate immunoassay platforms for malaria serology and for developing scalable serosurveillance strategies in elimination-phase settings.

## Author summary

Malaria elimination requires reliable serological tools to support surveillance activities in low-transmission settings. Serological assays that measure antibody responses are well-suited for this purpose. We aimed to provide direct, comparative evidence to help investigators and public health programs choose the appropriate assay for their specific needs. Using a single well-characterized malaria antigen, we systematically evaluated four common serological platforms—protein arrays, dot blot, ELISA, and Western blot—against an identical serum panel under standardized conditions. This approach enabled objective assessment of each method’s sensitivity, throughput, and practicality. Our results demonstrate that no single method is universally superior. Protein arrays offer the highest throughput with minimal sample consumption, making them ideal for large-scale seroepidemiological studies. ELISA provides the greatest analytical sensitivity for detecting low-level antibody responses. Dot blot may be useful as a simple and low-cost screening format, whereas Western blot is better suited for confirmatory laboratory analysis because it allows visualization of antigen-specific bands. This study provides a practical framework for selecting immunoassay platforms according to surveillance goals, sample availability, and resource constraints, and may support the development of scalable serosurveillance workflows in elimination-phase settings.

## Introduction

*Plasmodium vivax* remains a significant obstacle to global malaria elimination efforts, owing to its broad geographical distribution. In 2024, an estimated 282 million malaria cases occurred worldwide [1]. *P. vivax* accounted for approximately 3.5% of this global burden, corresponding to an estimated 9.9 million cases, it remains the predominant malaria species outside Africa [1]. A critical barrier to *P. vivax* elimination lies in its unique hypnozoite stage—dormant liver forms that evade current interventions and trigger recurrent infections, perpetuating hidden transmission through relapsing low-level parasitemia [2]. In addition, *P. vivax* preferentially invades reticulocytes and accumulates in organs such as the spleen [3–5] and bone marrow [6,7], resulting in chronically low peripheral parasitemia. These biological features complicate case detection, routine diagnostics such as microscopy and rapid diagnostic tests (RDTs) have reduced sensitivity for *P. vivax* [8,9]. Consequently, substantial numbers of infections remain undetected, posing a critical challenge to elimination programs.

Serological surveillance offers a complementary approach by detecting *Plasmodium*-specific antibodies which are durable biomarkers of past exposure. This is particularly valuable in low-transmission and seasonal settings where parasite-based diagnostics underperform [10,11]. Serology can help identify residual transmission hotspots and guide targeted interventions [12]. Unlike direct parasite detection, serology enables the characterization of transmission intensity over time and provides exposure information.

Immunoassays provide the technical basis for large-scale *P. vivax* serosurveillance. Based on antigen–antibody interactions, they are widely used for both protein quantification and serological studies [13]. Recent developments have led to multiple formats, each differing in sensitivity, throughput, and material requirements. Among the most common are enzyme-linked immunosorbent assay (ELISA), Western blot, dot blot, and protein arrays. However, few studies have directly compared their performance under standardized conditions.

The merozoite surface protein (MSP) family, and particularly the PvMSP1–42 fragment, is a key candidate in blood-stage vaccine development. During red blood cell invasion, PvMSP1–42 is cleaved into PvMSP1–33 and PvMSP1–19. ELISA-based assays using this antigen have proven effective in detecting specific antibodies in infected individuals [14,15]. Notably, anti-PvMSP1–42 antibodies can persist for months to years after parasite clearance, highlighting their value as serological markers of prior *P. vivax* exposure [16].

In this study, we conducted a direct comparison of four immunoassay platforms using a recombinant His-tagged PvMSP1–42 protein and sera from immunized mice, *P. vivax-*infected patients and healthy individuals. To optimize protein array performance, we also evaluated different surface chemistries (ion-coated, poly-L-lysine-coated, and APES-coated slides) and tested two diluents, phosphate-buffered saline (PBS) and Protein Microarray Spot Diluent (MSD). Our goal was not only to compare analytical performance but also to provide practical guidance for selecting appropriate serological tools for different research and surveillance applications. By highlighting trade-offs among sensitivity, dynamic range, throughput, and sample conservation, this work aims to support public health programs in implementing cost-effective serological surveillance for exposure monitoring and elimination-phase surveillance.

## Methods

### Ethics statement

The mice were obtained from the Experimental Animal Center of Yangzhou University, and all procedures were conducted in accordance with protocols approved by the Laboratory Animal Ethics Committee (protocol No. 202305004). Blood samples were collected after protocol approval by the Institutional Review Board (IRB00004221) of the Jiangsu Institute of Parasitic Disease in Wuxi, China and the Research Ethics Committee of the Affiliated Hospital of Yangzhou University, Yangzhou, China (YXYLL-2025-98). Written informed consent was obtained from each participant prior to the commencement of the study.

### Human serum samples

Human serum samples were obtained from individuals with acute *P. vivax* malaria (AVM, n = 66), acute *P. falciparum* malaria (AFM, n = 44), and healthy individuals (HI, n = 40). The AVM sera were collected from patients with acute uncomplicated malaria in Anhui Province, China, whereas the AFM sera were collected from patients with imported acute *falciparum* malaria diagnosed in China. All malaria infections were confirmed by microscopic examination and polymerase chain reaction (PCR), and only samples with confirmed single-species infections were included in the study. Healthy control samples were obtained from volunteers in Jiangsu Province, China, with no self-reported history of malaria. Available demographic and clinical characteristics of the study population are summarized in Table 1. All serum samples were aliquoted and stored at −80°C until use.

**Table 1:** Demographic and clinical characteristics of the study cohort.

Characteristics	AVM	AFM	HI
Total, n	66	44	40
Year of serum collection	2007	2025	2012, 2025
Parasitemia, median (IQR), parasites/μl	3,022 (1,141–6,479)	9,468 (1,075–60,350)	–
Sex, male/female, n	25/41	43/1	12/28
Age, mean ± SD, years	30 ± 12	45 ± 11	23 ± 5
Age, range, years	18-60	23–66	18–33

Abbreviations: AVM, Acute *P. vivax* infection; AFM, Acute *P. falciparum* infection; HI, Healthy individuals; IQR, interquartile range.

Information regarding the exact interval between symptom onset and blood collection, previous malaria episodes, and treatment status prior to sample collection was not consistently available for all archived samples and was therefore not included in the analysis. Asymptomatic malaria infections were not included in this study.

### Recombinant protein production and quantification

A recombinant His-tagged PvMSP1–42 protein was produced as previously described [16]. The purified protein concentration was determined using the Pierce Bicinchoninic Acid (BCA) Protein Assay Kit (Thermo Fisher Scientific, USA) [17] and adjusted to 1 mg/ml in PBS for stock preparation. For the assays, the protein was serially diluted from 200 ng/μl to 3.13 ng/μl.

In silico analysis using NetNGlyc, NetOGlyc, and NetPhos predicted several potential glycosylation and phosphorylation motifs. However, no experimentally validated post-translational modifications have been reported for the PvMSP1–42 fragment in *P. vivax*. Recombinant PvMSP1–42 contained His-tag, which was not removed prior to use in immunoassays. Quantification of tag-derived reactivity was performed by comparing the signal intensity of PvMSP1–42 with the mean intensity of unrelated His-tagged human proteins and a recombinant GST-tagged control protein expressed without a His-tag. These proteins were probed with sera from PvMSP1–42-immunized mice, healthy mice, *P. vivax*–infected patients and healthy individuals.

### Mouse serum collection

Female BALB/c mice aged 6–8 weeks were immunized subcutaneously three times with recombinant PvMSP1–42 protein or PBS (negative control). Sera were collected after the final immunization and serially diluted two-fold from 1:125 to 1:128,000 for serological analyses. Serial two-fold dilutions of immune mouse sera were analyzed across all four immunoassay platforms. Signal intensities were normalized to the value obtained at a 1:1000 dilution, which was defined as 1 arbitrary unit (AU) and used as a shared internal reference calibrator. Normalized titration curves were generated to determine the linear dynamic range for each platform. The overlapping linear region, defined as the dilution range showing proportional signal decline (R^2^ ≥ 0.99), was used to compare assay performance under harmonized analytical conditions.

### Use of protein microarray spot diluent (MSD)

To optimize protein spotting, the Protein Microarray Spot Diluent (MSD, CapitalBio Technology, China) was used. Proteins were initially diluted with PBS to double the final desired concentration. Subsequently, an equal volume of MSD was added to reach the final working concentration. MSD contains protein stabilizers and viscosity agents that enhance protein immobilization and reduce non-specific binding.

### Protein array fabrication and analysis

Three types of surface-modified glass slides were tested: ion-coated (Citotest Scientific, China), poly-L-lysine (PLL)-coated and APES-coated (Sangon Biotech, China). Serial dilutions of PvMSP1–42 protein (3.13–200 ng/μl) was spotted (1 μl per spot) onto the slides as previously described [16]. Blank controls (PBS) were included. After blocking with 5% BSA in PBST (PBS + 0.01% Tween-20) for 1 hour at 37°C, slides were incubated with anti-His-tag antibodies (1:100, Abmart, China) followed by Alexa Fluor 555 donkey anti-mouse IgG (1:100, Thermo Fisher Scientific). For serological assays, 1 μl of PvMSP1–42 (20 ng/μl) was spotted per well, and serially diluted mouse sera (from PvMSP1–42- or PBS-immunized mice) or human sera (1:10 diluted acute *P. vivax* infection or healthy individuals serum samples) as primary antibodies. Alexa Fluor 546 goat anti-human IgG (1:200, Invitrogen) was used as the secondary antibody for human sera.

Fluorescence signals were scanned using a LuxScan 10K-B microarray scanner (CapitalBio) and analyzed based on mean fluorescence intensity (MFI). Laser power and PMT settings were adjusted according to negative-control serum signals (mouse sera: laser 20, PMT 450–500; human sera: laser 5–10, PMT 450–500). The array layout consisted of 8 columns × 25 rows with a spot radius of 900 μm, horizontal spacing of 2408 μm, and vertical spacing of 2283 μm. Local median background subtraction and normalization to internal positive controls were applied. Intra- and inter-slide coefficients of variation (CV) were calculated as (SD/mean) × 100% to evaluate signal reproducibility across replicate spots and slides.

### Dot blot assay

Dot blot analysis was conducted using PVDF membranes cut into small circles and placed in a 24-well plate. The membranes were activated with methanol, rinsed three times with double-distilled water, and air-dried at room temperature until semi-dry (evidenced by the disappearance of the water film) prior to sample loading as previously described [18]. Three microliters of serially diluted PvMSP1–42 protein were spotted onto the membrane. After blocking overnight with 5% skimmed milk in TBST at 4°C, membranes were incubated with anti-His-tag antibodies (1:2,000, Huabio, China) overnight, followed by HRP-conjugated goat anti-mouse IgG (1:10,000, Thermo Fisher Scientific). Signals were visualized using the Tanon-5200 Chemiluminescent Imaging System (Tanon Science and Technology, China). For serological assays, membranes were incubated with diluted immunized sera or human sera (1:1000 dilution) instead of anti-His-tag antibodies.

### ELISA

For protein detection, 96-well ELISA plates (Nest Scientific, China) were coated overnight at 4°C with 100 μl/well of serially diluted PvMSP1–42 protein in carbonate-bicarbonate buffer (50 mM, pH 9.6) as described previously [19]. After blocking with 5% BSA in PBST for 1 hour at 37°C, wells were incubated with anti-His-tag antibodies (1:2,000, Huabio) for 2 hours at 37°C, followed by HRP-conjugated goat anti-mouse IgG (1:10,000, Thermo Fisher Scientific) for 1 hour. Colorimetric detection was performed using TMB substrate (Beyotime, China), and absorbance was measured at 620 nm using a microplate reader (Thermo Scientific Multiskan FC). For serological detection, plates were coated with PvMSP1–42 (5 ng/μl) and incubated with serially diluted immunized sera or human sera (1:1000 dilution) as primary antibodies.

ELISA reactions were performed using kinetic TMB substrate without stop solution, and absorbance was measured at 620 nm. Serially diluted mouse sera (1:62.5–1:128,000) and human sera (1:800–1:25,600) were monitored at 3, 4, 6, 8 and 10 minutes after substrate addition. The reaction rate (ΔOD/Δt) and regression coefficient (R^2^) were calculated between adjacent time points to identify the linear phase.

### Western blot

Serial dilutions of PvMSP1–42 protein were separated by 12% SDS-PAGE and transferred onto PVDF membranes (Millipore, USA), following the protocol described by Xu Q et al [20]. After blocking overnight at 4°C with 5% skimmed milk in TBST, membranes were incubated with anti-His-tag antibodies (1:2,000, Huabio) for 3 hours at room temperature, followed by HRP-conjugated goat anti-mouse IgG (1:10,000, Thermo Fisher Scientific) for 1 hour. Signals were developed using the BeyoECL Plus system and visualized with the Tanon-5200 imaging system. For serological detection, membranes were probed with diluted immunized sera or human sera (1:1000 dilution) instead of anti-His-tag antibodies.

### Statistical analysis

Data fitting was performed using five-parameter logistic (5PL) regression models [21]. Mean fluorescence intensities and OD values were analyzed using GraphPad Prism 8.4.2 (GraphPad Software, USA). To assess the reproducibility of duplicate spots, Pearson correlation coefficients were employed to quantify the linear agreement between replicate measurements. Unpaired two-tailed Student’s t-tests were used to verify the absence of significant mean biases between technical repeats, *p < 0.05, **p < 0.01, ***p < 0.001. Dynamic range and detection thresholds were determined based on 5PL model parameters.

Cutoff values of four immunoassay platforms were calculated as the mean value of the negative controls plus two standard deviations (mean + 2 SD) [22]. Some experiments were performed in independent batches, raw assay signals were normalized using batch-specific cutoff values and expressed as signal-to-cutoff (S/CO) ratios, S/CO = assay signal/batch-specific cutoff. This normalization approach was applied to protein array, ELISA, dot blot, and Western blot data. Samples with S/CO > 1 were considered positive, whereas samples with S/CO ≤ 1 were considered negative. S/CO values were subsequently used for visualization, correlation analyses, receiver operating characteristic (ROC) analyses, and calculations of sensitivity and specificity.

Spearman correlations were used to evaluate consistency across normalized results obtained from different assay methods. Statistical analyses were conducted using MedCalc Statistical Software (v22.009; https://www.medcalc.org/calc/diagnostic_test.php) for diagnostic test evaluations. Inter-method agreement was assessed via Cohen’s kappa coefficient (κ), calculated using GraphPad Prism QuickCalcs (https://www.graphpad.com/ quickcalcs/kappa1/). ROC analyses were conducted using Python web platform (https://toolshu.com/python3) applying the DeLong method to estimate the area under the curve (AUC) with 95% confidence intervals.

## Results

### Protein and antibody detection by protein arrays

Three types of surface-modified slides were evaluated for protein array performance: ion-modified, poly-L-lysine (PLL)-modified, and APES-modified slides.

**Fig 1:**
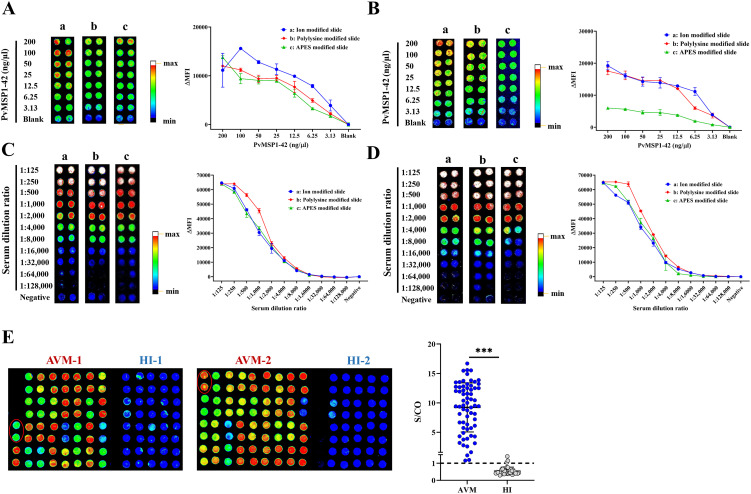
Detection of the concentration and specific antibodies against PvMSP1-42 by protein arrays. **(A–B)** Series of two-fold dilutions of PvMSP1-42 (3.13–200 ng/μl) probed with anti-His-tag antibody. ΔMFI = MFI (diluted protein) − MFI (blank). **(C–D)** Series of two-fold dilutions of sera (1:125 to 1:128,000) probed with spotted PvMSP1-42. ΔMFI = MFI (diluted serum) − MFI (negative). PvMSP1-42 was spotted onto three different substrate-modified slides. In **(A and C)**, proteins were diluted in PBS; in (**B** and **D**) in MSD. **(E)** Representative protein array images showing anti-PvMSP1-42 IgG reactivity in sera from acute *P. vivax* infection (AVM) and healthy individuals (HI). The right panel shows the corresponding quantitative analysis. Signal intensities were normalized using batch-specific cutoff values and expressed as signal-to-cutoff (S/CO) ratios (S/CO = MFI/cutoff value). The dashed line indicates the positivity threshold (S/CO = 1).

Using anti-His-tag antibodies, all slides showed a dose-dependent decrease in mean fluorescence intensity (MFI) with increasing protein dilution (Fig 1A–B). Detection thresholds reached 3.13 ng/μl for protein arrays using MSD as diluent. PLL-modified slides exhibited the highest linearity (R^2^ = 0.98) and the broadest dynamic range (Panels A–B of Fig A and Table A in S1 Appendix). For serological detection, strong signals were observed with immunized sera, and positive detection was maintained up to a serum dilution of 1:32,000 across all three surface-modified slides and both diluent conditions (Fig 1C–D). Notably, the combination of PLL-modified slides and MSD achieved detection up to 1:64,000 serum dilution, outperforming ion- and APES-modified slides (Panels C–D of Fig A and Table B in S1 Appendix). Subsequently, sera from 66 acute *P. vivax*-infected patients and 40 healthy individuals were tested using PLL-modified arrays. Because the healthy control sera were analyzed in two independent batches, background reactivity was first compared between batches. No substantial difference was observed, supporting the use of batch-specific cutoff normalization and pooled S/CO-based analyses (Fig B in S1 Appendix). The mean S/CO value was significantly higher in patient samples (9.23 ± 0.51) than in healthy controls (0.55 ± 0.04). Using microscopy- and PCR-confirmed single-species *P. vivax* infection status as the reference standard, the protein array assay achieved 100% sensitivity and 93% specificity and showed excellent agreement with the reference infection status, with a Cohen’s κ value of 0.93 (95% CI: 0.87–1.00) (Fig 1E, Table 2). Pearson correlation analyses confirmed excellent reproducibility across replicates (R^2^ > 0.82, *p* < 0.001; Fig C in S1 Appendix). Signal variation analysis showed a mean intra-slide CV of 9.3% and an inter-slide CV of 5.6%.

**Table 2:** Sensitivity and specificity of IgG antibodies against PvMSP1-42 in serum samples using four different methods.

	Sample	No. positive	No. negative	Sensitivity ^1^ (95% CI)	Specificity ^2^ (95% CI)	Signal-to-cutoff value		κ^3^ (95%CI)	SE of κ
						mean ± SEM	Highest Lowest		
**Protein arrays**	AVM	66	0	100% (95–100%)		9.23 ± 0.51	16.68	0.93 (0.87–1.00)	0.04
							1.16		
	HI	3	37		93% (80–98%)	0.55 ± 0.04	1.40		
							0.29		
**Dot blot**	AVM	53	13	80% (69–89%)		2.28 ± 0.16	5.39	0.71 (0.58–0.85)	0.06
							0.31		
	HI	2	38		95% (83–99%)	0.49 ± 0.04	1.47		
							0.22		
**ELISA**	AVM	62	4	94% (85–98%)		5.32 ± 0.26	7.79	0.88 (0.79–0.97)	0.05
							0.59		
	HI	2	38		95% (83–99%)	0.62 ± 0.03	1.20		
							0.38		
**Western blot**	AVM	62	4	94% (85–98%)		2.74 ± 0.18	6.11	0.86 (0.76–0.96)	0.05
							0.37		
	HI	3	37		93% (80–98%)	0.51 ± 0.04	1.22		
							0.17		

^1^Sensitivity = Positive serum number/Patient serum number×100%

^2^Specificity = Negative serum number/healthy individual serum number×100%

^3^The κ values in this table indicate the agreement between each immunoassay platform and the PCR-confirmed infection status.

Abbreviations: CI, confidence interval; SEM, standard error of the mean; κ, Kappa; SE, standard error; AVM, acute *P. vivax* infection; HI, healthy individuals.

In PvMSP1–42–immunized mice, all His-tagged proteins exhibited stronger signals than in healthy controls, whereas the GST-tagged control remained unchanged (Panel A of Fig D in S1 Appendix). In human sera, unrelated His-tagged human proteins showed no significant reactivity with either *P. vivax* patient or healthy control samples (Panel B of Fig D in S1 Appendix). The His-tag–derived component accounted for only ~12–30% of the total signal in mouse sera, as quantified by comparing His–PvMSP1–42 with the mean intensity of unrelated His-tagged proteins (Panel C of Fig D in S1 Appendix). This component represents a stable background rather than antigen-specific binding. These results demonstrate that His-tag–related reactivity is confined to the murine immunization setting and represents only a minor, constant background (< 30%) without affecting dynamic range or antigen specificity. These findings indicate that the reactivity observed in human sera was not attributable to His-tag recognition [8,23,24].

### Performance of dot blot assay

**Fig 2:**
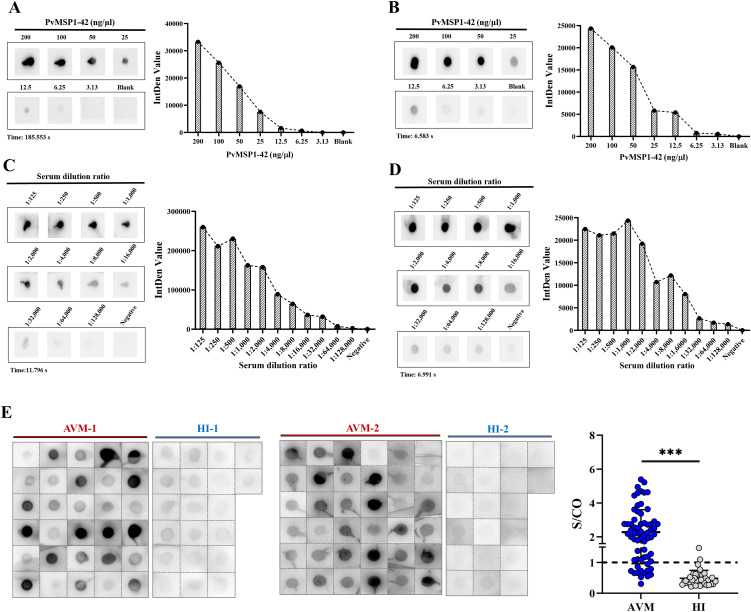
Detection of PvMSP1-42 by dot blot assay. **(A–B)** Detection of various protein concentrations using constant primary and secondary antibodies. **(C–D)** Detection of sera at serial dilutions with a fixed PvMSP1-42 concentration. **(E)** Anti-PvMSP1-42 IgG detection in AVM and HI sera by dot blot. Data are presented as signal-to-cutoff (S/CO) ratios, calculated as IntDen value divided by the corresponding cutoff value. The dashed line indicates the positivity threshold (S/CO = 1). Proteins in (**A, C, E**) were diluted in PBS, those in (**B, D**) were diluted in MSD. Original uncropped images of the dot blot experiments are provided in S1 Raw Gel.

Dot blot assays using PVDF membranes showed consistent performance across protein and serum dilutions. The detection threshold was 6.25 ng/μl with PBS and improved to 3.13 ng/μl using MSD (Fig 2A–B). For serological assays, positive signals were detected at serum dilutions up to 1:128,000 when using MSD, compared to 1:64,000 with PBS (Fig 2C–D). Dilution-curve analysis showed that signal saturation occurred at serum dilutions of 1:500 or lower (Fig 2D). Sensitivity for low-concentration proteins and sera was enhanced with MSD, although dot blot tended to saturate at high serum concentrations (Table C in S1 Appendix).

The dot blot assay using PBS-diluted PvMSP1–42 protein showed relatively lower sensitivity than the other platforms in detecting *P. vivax*-specific antibodies in clinical serum samples. Compared with the reference infection status, 13 *P. vivax*-positive samples were not detected by dot blot, resulting in a sensitivity of 80% (95% CI: 69–89%) and a Cohen’s κ value of 0.71 (95% CI: 0.58–0.85). However, the assay maintained good specificity, reaching 95% (95% CI: 83–99%) (Fig 2E and Table 2).

### ELISA sensitivity and detection range

**Fig 3:**
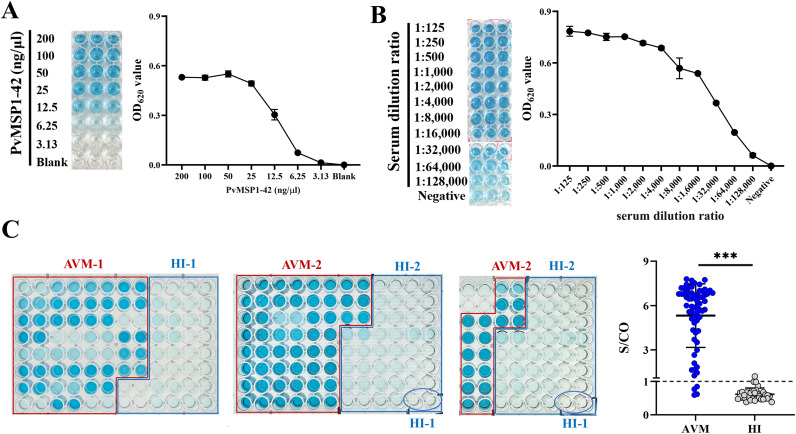
Detection of PvMSP1-42 by ELISA. **(A)** Detection of serial dilutions of PvMSP1-42 with anti-His-tag antibody. **(B)** Detection of serially diluted sera using 5 ng/μl of PvMSP1-42. OD_620_ values were corrected by subtracting blank or negative controls. **(C)** Anti-PvMSP1-42 IgG levels in AVM and HI sera. Data are shown as signal-to-cutoff (S/CO) ratios = OD_620_ value/cutoff value.

PvMSP1–42 protein detection was linear between 6.25 ng/μl and 50 ng/μl, with signal saturation observed at 50 ng/μl (Fig 3A). For serological assays, strong reactivity was maintained even at a serum dilution of 1:128,000 (Fig 3B). The ELISA also showed high diagnostic performance in detecting *P. vivax*-specific antibodies, with a sensitivity of 94% (95% CI: 85–98%) and a specificity of 95% (95% CI: 83–99%). Consistently, ELISA showed excellent agreement with the reference infection status, with a Cohen’s κ value of 0.88 (95% CI: 0.79–0.97) (Fig 3C and Table 2). Duplicate wells showed high consistency (R^2^ = 0.99, Fig E in S1 Appendix).

ELISA kinetics were evaluated from 3 to 10 minutes after TMB addition for both mouse and human sera (Fig F in S1 Appendix). Signals from mouse sera remained within the linear range throughout the evaluated interval. For human sera, the signal plateaued after 6 minutes at lower dilutions (1:800–1:1,600), whereas dilutions from 1:3,200–1:25,600 remained within the linear range. Based on these kinetic profiles, all ELISA readings were standardized to 5 minutes after TMB addition, which was within the linear phase for both sample types.

### Protein and antibody detection by Western blot

**Fig 4:**
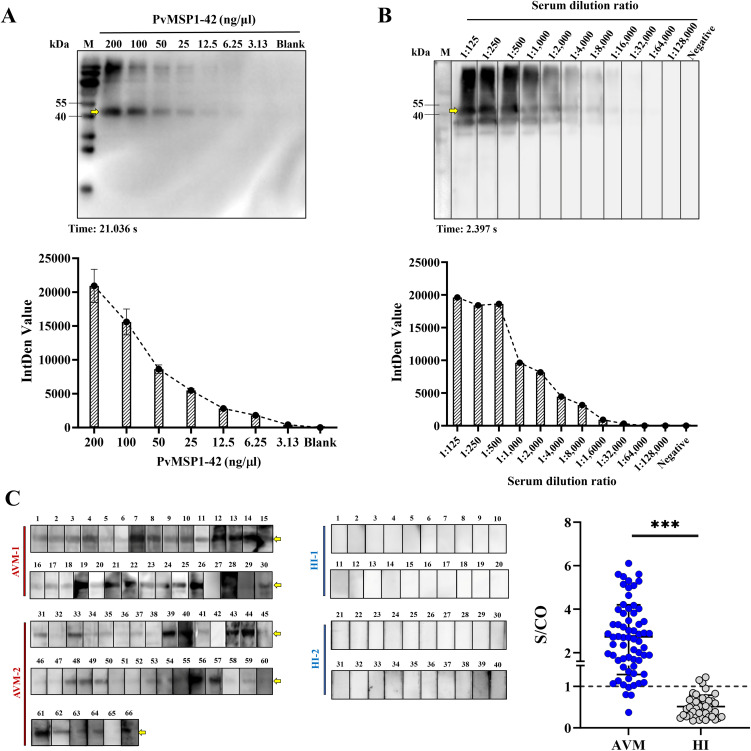
Detection of PvMSP1-42 by Western blot. **(A)** Detection of serial dilutions of PvMSP1-42 using anti-His-tag antibody. **(B)** Detection of serially diluted sera using a fixed PvMSP1-42 amount. Signal intensities were corrected by subtraction of blank or negative controls. **(C)** Detection of anti-PvMSP1-42 IgG in AVM and HI sera by Western blot. Data are shown as signal-to-cutoff (S/CO) ratios = IntDen value/ cutoff value. The yellow arrow indicates expected ~42 kDa band corresponding to PvMSP1-42. Original uncropped images of the Western blot experiments are provided in S1 Raw Gel.

Western blot detected PvMSP1–42 protein down to 6.25 ng/μl (Fig 4A). No saturation was observed at high protein concentrations (200 ng/μl). For serological detection, positive signals were observed up to a 1:32,000 serum dilution, with signal saturation at 1:500 (Fig 4B). In clinical serum samples, PvMSP1–42-specific reactivity was mainly observed around the expected molecular weight, although weak background or nonspecific signals were present in some lanes (Fig 4C). Compared with the reference infection status, Western blot analysis showed 94% sensitivity (95% CI: 85–98%) and 93% specificity (95% CI: 80–98%), with four patient samples showing undetectable antibody reactivity and three healthy individual samples showing nonspecific antibody reactivity. The assay showed excellent agreement with the reference infection status, with a Cohen’s κ value of 0.86 (95% CI: 0.76–0.96) (Fig 4C and Table 2).

### Assessment of cross-reactivity with *P. falciparum* sera

To further evaluate potential species cross-reactivity, sera from patients with acute *P. falciparum* malaria (AFM) were tested against PvMSP1–42 using protein arrays and ELISA. Cross-reactive responses were detected in a subset of AFM samples by both platforms, with positivity rates of 36% and 34%, respectively (Panels A–B of Fig G and Table D in S1 Appendix). Although most AFM samples showed lower signal intensities than acute *P. vivax* infection samples, a subset exceeded the positivity threshold in both protein arrays and ELISA, indicating detectable cross-reactivity to PvMSP1–42. Based on the protein array and ELISA results, we selected a representative subset of 16 AFM serum samples for qualitative confirmation by dot blot and Western blot, including 7 samples with strong reactivity, 5 with moderate reactivity, and 4 with weak reactivity against PvMSP1–42. Although several AFM samples showed weak reactivity, the signals were generally less pronounced than those observed in acute *P. vivax* infection and did not produce a clear discriminatory pattern (Panels C–D of Fig G in S1 Appendix).

### Comparison of the four immunoassay methods

To harmonize antibody detection across platforms, matched titration curves were established using serially diluted immune mouse sera (Fig H in S1 Appendix). When signal intensities were expressed relative to 1 AU at a 1:1000 dilution, all four immunoassay platforms displayed a common linear dynamic range between 1:1000 and 1:16,000. Within this range, signal intensities decreased proportionally with serum dilution, indicating comparable linearity and quantification behavior. At lower dilutions, signal saturation was observed in array- and blot-based assays, whereas at higher dilutions, signal loss became more pronounced in ELISA and Western blot. These findings demonstrate cross-platform consistency after normalization.

**Fig 5:**
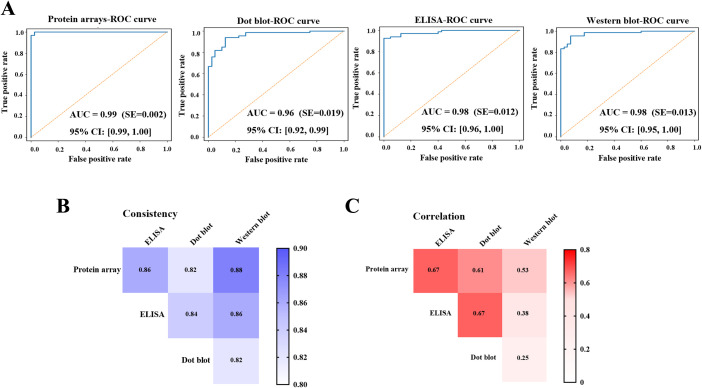
Comparative evaluation of four methods for *P. vivax* infection using PvMSP1-42 antigen. **(A)** Receiver operating characteristic (ROC) curve analysis. **(B)** Pairwise concordance among the four platforms based on binary positive/negative classifications. The color bar (0.80–0.90) indicates Cohen’s κ values. **(C)** Pairwise correlation among methods based on continuous signal-to-cutoff values. The color bar (0.00–0.80) indicates Spearman correlation coefficients.

Protein arrays demonstrated the broadest dynamic range while requiring the lowest amounts of protein and antibodies. Protein arrays, ELISA, and Western blot all showed excellent diagnostic performance (AUC = 0.98–0.99), whereas dot blot displayed slightly lower but still strong discriminatory ability (AUC = 0.96) (Fig 5A). Dot blot assays exhibited enhanced sensitivity when using MSD as a diluent, although they showed a tendency to saturate at high serum concentrations. ELISA offered the highest sensitivity for detecting low antibody titers but experienced saturation at higher antigen concentrations. Western blot allowed direct visualization of antigen-specific bands, thereby providing qualitative confirmation of antibody recognition. While its sensitivity and specificity were comparable to those of ELISA, the assay required substantially higher protein input and longer processing times, limiting its suitability for large-scale serological screening. In addition, we visualized the raw signal distributions for each group using violin plots and histograms, illustrating clear separation between positive (AVM) and negative (HI) samples and confirming that the chosen thresholds align with the underlying score distributions (Fig I in S1 Appendix).

**Fig 6:**
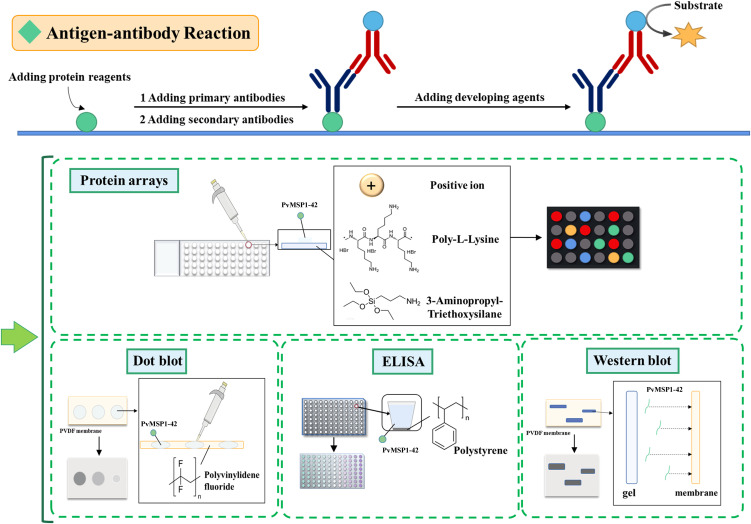
Schematic overview of the four immunoassay techniques. Overview of protein arrays, dot blot, ELISA, and Western blot workflows. The schematic outlines key procedural steps and substrate types, highlighting comparative sensitivity and field applicability.

Agreement analysis further showed high pairwise consistency among the four platforms, with Cohen’s κ values ranging from 0.82 to 0.88 (Fig 5B). In contrast, Spearman correlation coefficients ranged from weak to moderate (r = 0.25–0.67) (Fig 5C). This difference indicates that the platforms generally classified samples similarly as positive or negative, but the magnitude of quantitative signals was not fully interchangeable across assay formats. The strongest pairwise agreement was observed between protein array and Western blot (κ = 0.88), whereas the highest correlations were observed between ELISA and protein arrays, and between ELISA and dot blot (r = 0.67). A direct comparison of the four platforms highlighted their distinct analytical and operational characteristics (Fig 6 and Table 3).

**Table 3:** Comparative performance characteristics of the four immunoassay platforms. Summary of detection thresholds, dynamic ranges, sensitivity for low antibody titers, protein and antibody consumption, and assay complexity for protein arrays, dot blot, ELISA, and Western blot.

		Protein arrays		Dot blot		ELISA	Western blot
**Principle**		Antigen-antibody reaction					
**Sample carrier**		Slides with different modifications		PVDF or NC membrane		Polystyrene microreaction plates	PVDF or NC membrane
**Protein diluent**		PBS ^**1**^ or MSD ^**2**^		PBS or MSD		Coating buffer ^**3**^	PBS
**Detection Equipment**		Microarray scanner		Luminescence imaging system		Microplate reader	Luminescence imaging system
**Actual protein input** ^**4**^		20 ng/μl 20 ng/spot (1 μl)		200 ng/μl 600 ng/spot (3 μl)		5 ng/μl 500 ng/well (100 μl)	200 ng/μl 1000 ng/lane (5 μl)
**Actual serum input** ^**5**^		1:10 0.1 μl/spot (1 μl)		1:1,000 0.5 μl/spot (500 μl)		1:1,000 0.1 μl/well (100 μl)	1:1,000 1 μl/lane (1,000 μl)
**Ranges of protein quantification**		3.13-200 ng/μl	3.13-200 ng/μl	6.25-200 ng/μl	3.13-200 ng/μl	6.25-50 ng/μl	6.25-200 ng/μl
**Ranges of serological detection**		1:125-1:32,000 dilution	1:125-1:64,000 Dilution ^**6**^	1:500-1:64,000 dilution	1:1,000-1:128,000 dilution	1:250-1:128,000 dilution	1:500-1:32,000 dilution
**Characteristics**	**Throughput**	High (More than 160 samples per slide)		Medium (More than 20 samples per membrane ^**7**^)		Medium (96 samples per plates)	Low (15 samples per membrane ^**8**^)
	**Time required** ^**9**^	1 ~ 2 h (37 °C)		10-30 min (RT)		1 ~ 2 h (37 °C)	3 ~ 4 h
**Cost**	**Reagents and consumables** ^**10**^	Starting at ~$5		Starting at ~$8		Starting at ~$20	Starting at ~$10
	**Detection Equipment**	Starting at ~$25,000		Starting at ~$20,000		Starting at ~$8,000	Starting at ~$20,000
**Optimal Application Scenario**		• High-throughput serosurveillance • Multiplex immune profiling • Serological exposure markers discovery		• Field-based preliminary screening • Resource-limited surveillance settings		• Quantitative antibody detection • Longitudinal cohort studies	• Antigen specificity validation • Confirmatory diagnostic testing

^1^PBS: Phosphate-buffer saline, pH 8.0.

^2^MSD: Protein Microarray Spot Diluent.

^3^Coating buffer: 50 mM sodium carbonate–sodium bicarbonate buffer, pH 9.6.

^4^Protein dosage used for serological detection, expressed as concentration (ng/μl) and total mass per spot or well.

^5^Serum volume used for antigen detection, expressed as dilution factor and absolute sample volume per spot or well.

^6^Antibody detection extended to a serum dilution of 1:64,000 on PLL-modified slides when PvMSP1–42 was prepared in MSD, ion- and APES-modified slides reached 1:32,000.

^7^The size of each PVDF/NC membrane is 6.6 × 8.5 cm. The size of the dot blot spots is not fixed, in this experiment, the size is approximately 0.7 × 0.7 cm, and the spacing is about 1.0 cm.

^8^The size of each PVDF/NC membrane is 6.6 × 8.5 cm.

^9^Protein fixation time.

^10^Including sample carrier, primary/secondary antibody and developer fluid. Western blot also included SDS-PAGE gel.

## Discussion

Recent advances in malaria serology have mainly focused on antigen discovery, antibody kinetics, and the development of serological markers for estimating recent or past exposure. Previous studies have shown that selected antibody responses can reflect recent changes in malaria transmission at the population level, and that interpretation of *P. vivax* serological signals depends strongly on antigen-specific antibody longevity and transmission intensity [25,26]. More recent work has further developed validated antigen panels for identifying recent *P. vivax* infection [27,28] and explored serological test-and-treat strategies as potential tools for elimination settings [29]. These studies highlight the growing importance of serology in malaria surveillance, particularly in low-transmission areas where submicroscopic or clinically silent infections may not be fully captured by routine parasitological diagnosis. However, the practical implementation of serological surveillance depends not only on antigen selection and antibody kinetics, but also on the analytical platform used to measure antibody responses. Our comparative analysis demonstrated that while all four immunoassay platforms (protein arrays, dot blot, ELISA and Western blot) are capable of detecting *P. vivax*-specific antibodies and antigens with high reproducibility, their diagnostic and analytical performances differ markedly.

Full standardization of antigen or antibody concentrations across different immunoassays is not easy due to platform-specific optimal conditions. To enable cross-platform comparability, we employed a standardized normalization approach using titrated mouse immune sera. This strategy demonstrated robust inter-assay consistency and maintained proportional dose-response relationships under normalized experimental conditions. Among the evaluated platforms, protein arrays showed the broadest dynamic range (3.13–200 ng/μl), required minimal amounts of protein and antibody, and yielded the highest diagnostic accuracy, achieving 100% sensitivity, 93% specificity, and an area under the curve (AUC) of 0.99. These results are in line with previous studies highlighting the multiplexing potential and high-throughput capacity of protein arrays in serological surveillance [30,31]. Furthermore, the improved signal intensity and reproducibility observed with poly-L-lysine (PLL)-coated slides underscore the importance of optimizing slide surface chemistry in enhancing assay sensitivity. Further innovations in surface modifications, such as nanostructured or functionalized substrates, may improve the signal-to-noise ratio and enable more reliable detection of low-abundance antibody responses [32–36].

Dot blot assays, while relatively less sensitive (80%), offered simplicity, cost-effectiveness [37], and operational feasibility [38], particularly when using a protein-stabilizing diluent (MSD). MSD improved dot morphology and signal consistency, thereby enhancing detection at low serum dilutions and making the method more suitable for field deployment. However, dot blot’s vulnerability to signal saturation at high antibody levels [39] and its lower diagnostic accuracy limit its utility for confirmatory testing [40]. Nonetheless, dot blot may serve as a rapid, low-resource preliminary screen in endemic areas where high-throughput, low-cost detection is required [18,41,42].

ELISA remained the most sensitive platform for detecting low antibody titers, identifying specific responses at serum dilutions up to 1:128,000. This high sensitivity makes ELISA particularly valuable in low-endemic or elimination-phase settings, where serological tools can provide complementary information on recent or past exposure [43–46]. Although the signal plateaued at high antigen concentrations, ELISA’s quantitative output, standardizability, and scalability maintain its role as the gold standard in seroepidemiological studies [43–46].

Western blot offered high specificity and the unique advantage of molecular weight resolution, allowing confirmation of antigen identity and antibody reactivity profiles [47–49]. Despite requiring greater protein input, longer assay time, and higher technical expertise, Western blot remains a valuable reference method for serological confirmation or antigen differentiation in research and diagnostic settings [50].

Across platforms, moderate correlations were observed between ELISA and protein arrays (r = 0.67) and between ELISA and dot blot (r = 0.67), indicating broadly comparable patterns of antibody detection despite methodological differences. Although all continuous readouts were normalized as signal-to-cutoff ratios, Spearman correlations between platforms remained weak to moderate, suggesting that S/CO normalization improved cross-platform comparability but did not make signal magnitudes directly interchangeable. This is reasonable because kappa evaluates binary positive/negative agreement, whereas Spearman correlation reflects the ranking of continuous signal values. Therefore, the platforms may classify samples similarly while still differing in quantitative signal intensity due to differences in antigen presentation, signal amplification, dynamic range, background correction, and image-based quantification. The lower correlation between dot blot and Western blot (r = 0.25) may reflect differences in assay format and signal quantification. The discrepancy between dot blot and Western blot results may be partly explained by differences in epitope presentation and assay format, dot blot recognizes conformational epitopes in native protein structures, while Western blot detects linear epitopes [51]. The high-density antigen spotting in dot blot enhances sensitivity to low-affinity antibodies, whereas Western blot requires higher-affinity binding [23]. Additionally, nonspecific binding from serum components like lipoproteins is more pronounced in dot blot due to matrix interference, while SDS treatment in Western blot reduces such background noise [52]. Excellent agreement between ELISA and Western blot (κ = 0.86) supports Western blot as a reference confirmatory assay despite its semi-quantitative nature.

Together, our findings provide practical guidance for selecting immunoassay platforms according to analytical objectives, sample constraints and resource availability. Protein arrays were particularly suitable for high-throughput surveillance studies requiring low input and multiplex capacity. ELISA was optimal for detecting low-level antibody responses in seroepidemiological research, while dot blot and Western blot served as complementary tools with dot blot for rapid screening, settings prioritizing cost, simplicity and Western blot for confirmatory specificity. Notably, our results highlight that minor methodological adjustments, such as buffer composition or surface chemistry, can significantly improve assay performance [44,46]. Such refinements are essential for adapting serological tools to diverse research and field applications. The capacity of protein arrays to simultaneously measure responses to multiple antigens further positions them as a promising platform for biomarker discovery and multiplex serological profiling in malaria-endemic populations [53–55]. Although cross-reactivity with antibodies against *P. falciparum* antigens cannot be excluded due to the partial sequence conservation of PvMSP1–42, such reactivity should be interpreted cautiously, particularly in co-endemic settings. In the context of elimination programs, this broader detection is operationally advantageous, as it helps to identify areas of recent malaria transmission—regardless of species—and guide targeted surveillance and intervention strategies [56].

Although this study systematically compared four major immunoassay techniques, several limitations should be acknowledged. First, our analysis focused on a single recombinant antigen, PvMSP1–42. Second, although we included *P. falciparum*-infected sera to assess potential cross-reactivity, further validation in co-endemic settings, mixed-infection samples, and individuals with other parasitic infections is still required to better define the real-world specificity of PvMSP1–42-based detection. Third, the clinical cohort remained relatively limited, and longitudinal samples were not available to evaluate antibody titer decay or transmission dynamics. Future research should expand comparative evaluations to a broader panel of *Plasmodium* antigens, across larger and geographically diverse cohorts. Integration of novel capture chemistries and digital signal detection may further enhance sensitivity and field deployability. Such approaches, combined with travel history data and molecular diagnostics, could improve hotspot identification in highly mobile populations and enable precision targeting of interventions.

By systematically benchmarking four major immunoassay platforms under standardized conditions, this study fills an important evidence gap and provides a framework for evidence-based assay selection. These findings may guide rational design of scalable serosurveillance systems to support elimination-phase surveillance.

## Conclusions

This study provides a comprehensive comparison of four widely used immunoassay platforms using a recombinant PvMSP1–42 protein, immunized mouse sera and patients’ sera with *P. vivax* infection. Protein arrays emerged as an efficient method, offering an excellent balance between sensitivity, dynamic range, throughput, and sample conservation, while ELISA demonstrated superior sensitivity for detecting low antibody titers. Dot blot and Western blot also provided valuable complementary approaches depending on specific research needs. These findings offer practical guidance for selecting appropriate immunoassay strategies and highlight the importance of methodological optimizations to enhance detection performance. Future work should expand these comparisons to larger clinical cohorts and a broader range of antigens to fully leverage the potential of immunoassay technologies.

## Supporting information

S1 Appendix Supplementary figures and tables. (PDF)

S1 Raw Gel Original images of WB and Dot blot. (XLSX)
