# Supplementary material for: Comparative evaluation of four immunoassays for Plasmodium vivax serological detection using recombinant PvMSP1-42 antigen
Source: PLoS Negl Trop Dis. 2026 Aug 5;20(8):e0014570. doi: 10.1371/journal.pntd.0014570 (PMC13440851; doi:10.1371/journal.pntd.0014570)
Supplement: Supplementary file 1 [file pntd.0014570.s001.pdf]

S1 Appendix

1 Supplementary Figures and Tables

1.1 Supplementary Figures

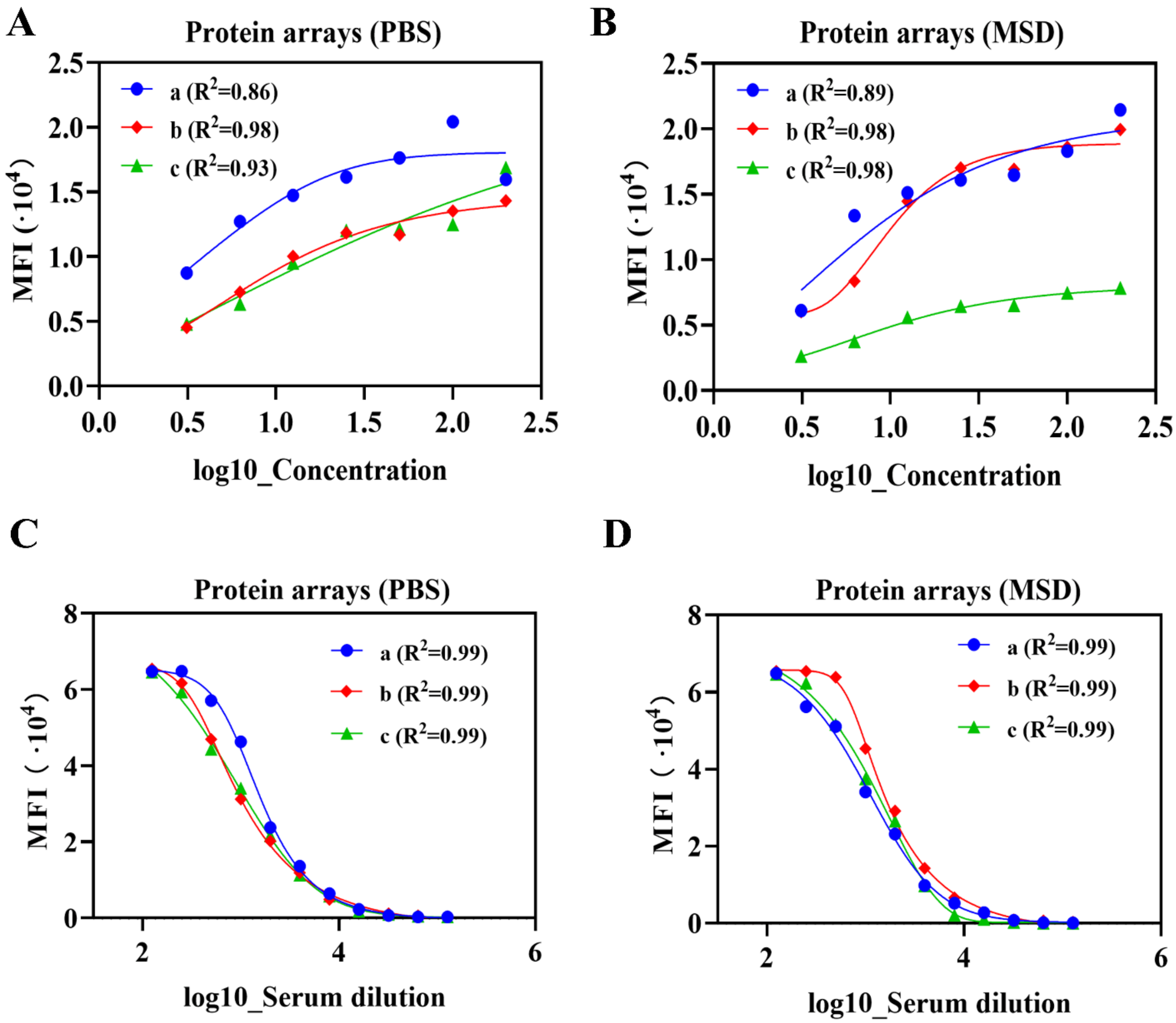

Fig A. Five-parameter logistic fitting curves of the three modified slides. (**A-B**) Fitted curves generated from serial two-fold dilutions of recombinant PvMSP1-42 protein (3.13 -200 ng/μL). (**C-D**) Fitted curves based on serial dilutions of sera (1:125 to 1:128,000). In (**A)** and (**C),** protein and serum samples were diluted in PBS; in (**B)** and (**D),** in MSD. Each panel includes data from the three substrate types: (a) ion-modified slide, (b) PLL-modified slide, (c) APES-modified slide.

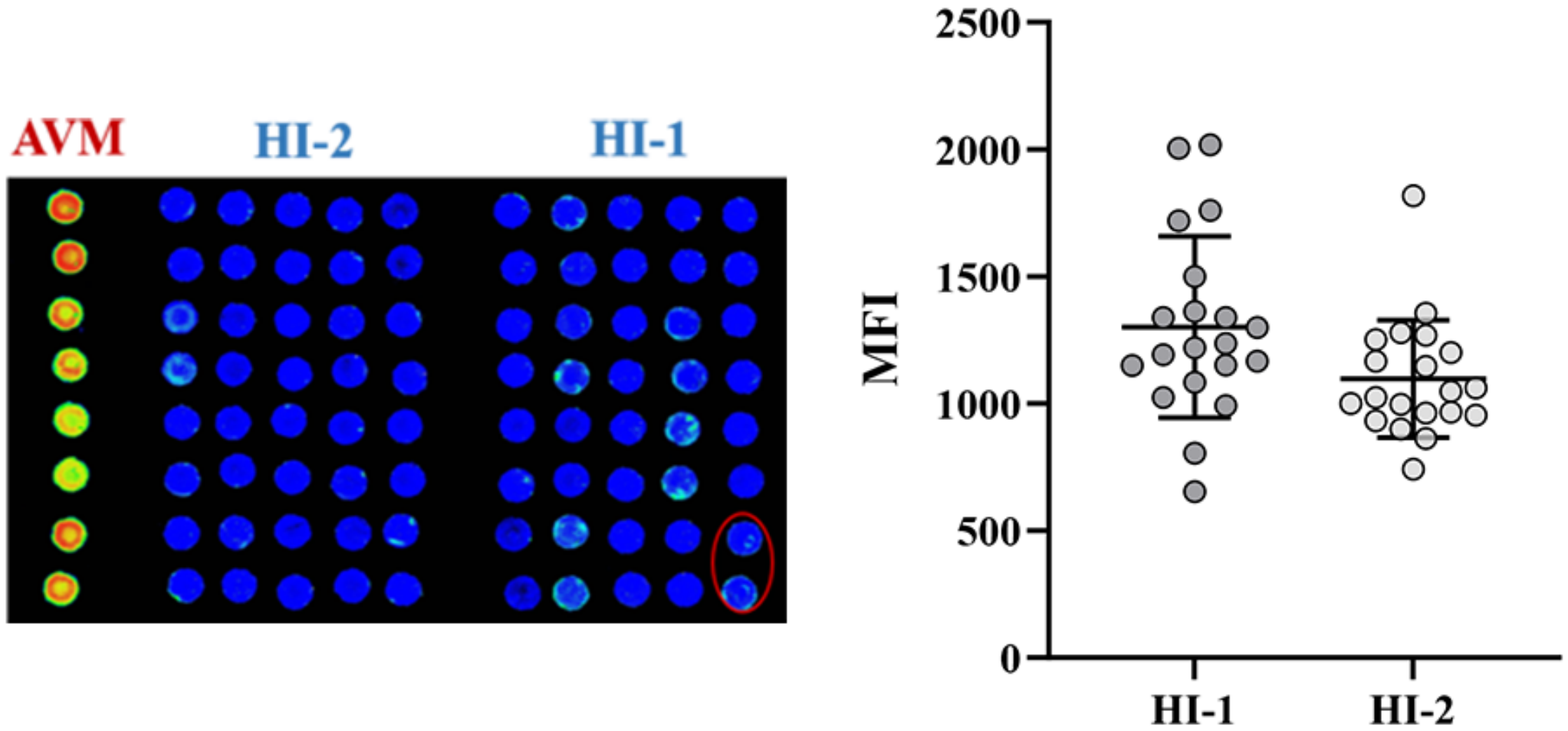

Fig B. Comparison of background reactivity between two independent batches of healthy control sera (HI-1 and HI-2) in the protein array assay. Representative array images and corresponding mean fluorescence intensity (MFI) values are shown. Each serum sample was analyzed in duplicate, and the red circles indicate representative paired replicate spots.

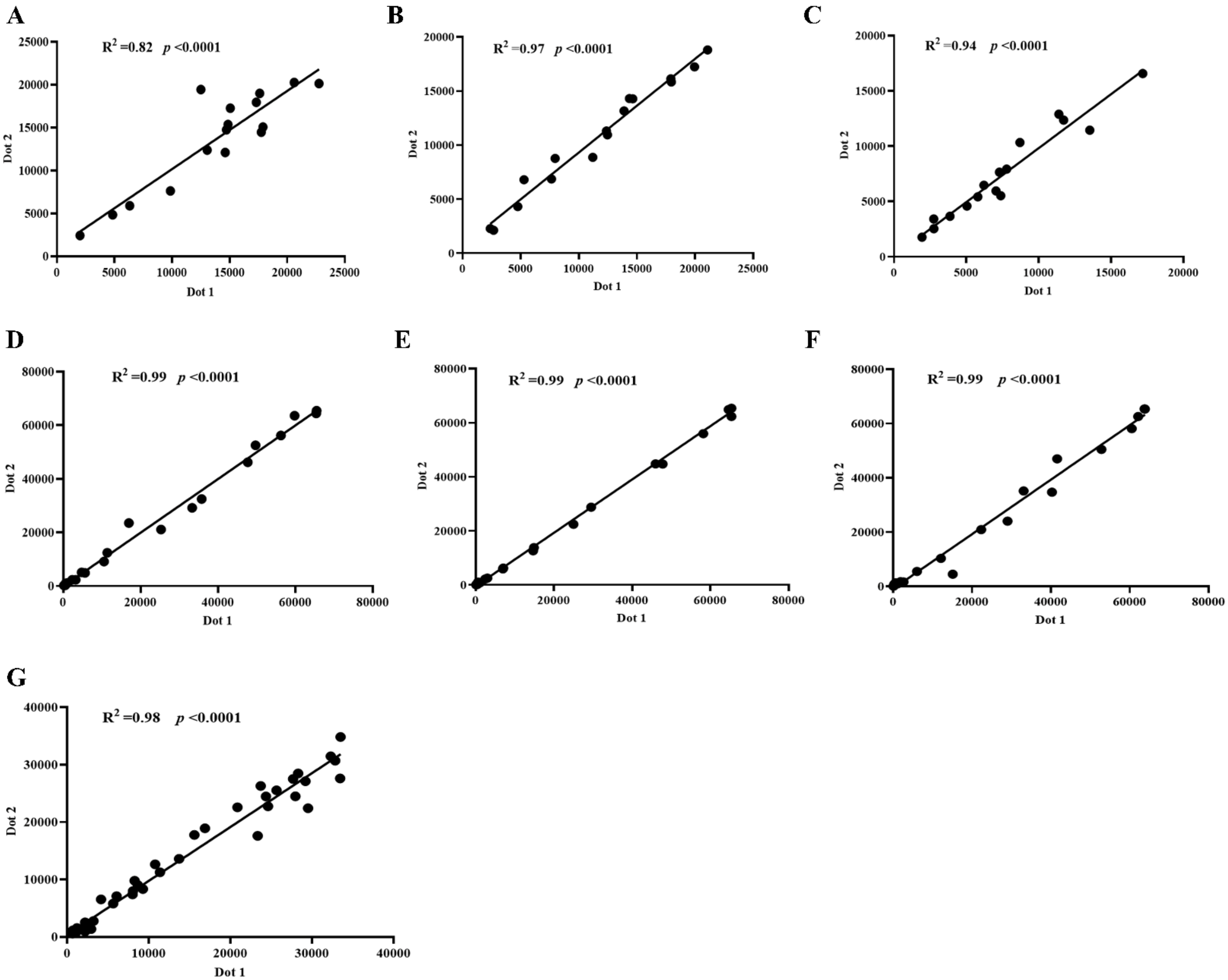

Fig C. Reproducibility of duplicate protein-array spots on different surface-modified slides. Pearson correlation analyses were performed to assess reproducibility between duplicate protein spots on ion-modified **(A and D)**, poly-L-lysine (PLL)-modified **(B, E and G)**, and APES-modified slides **(C and F)**.

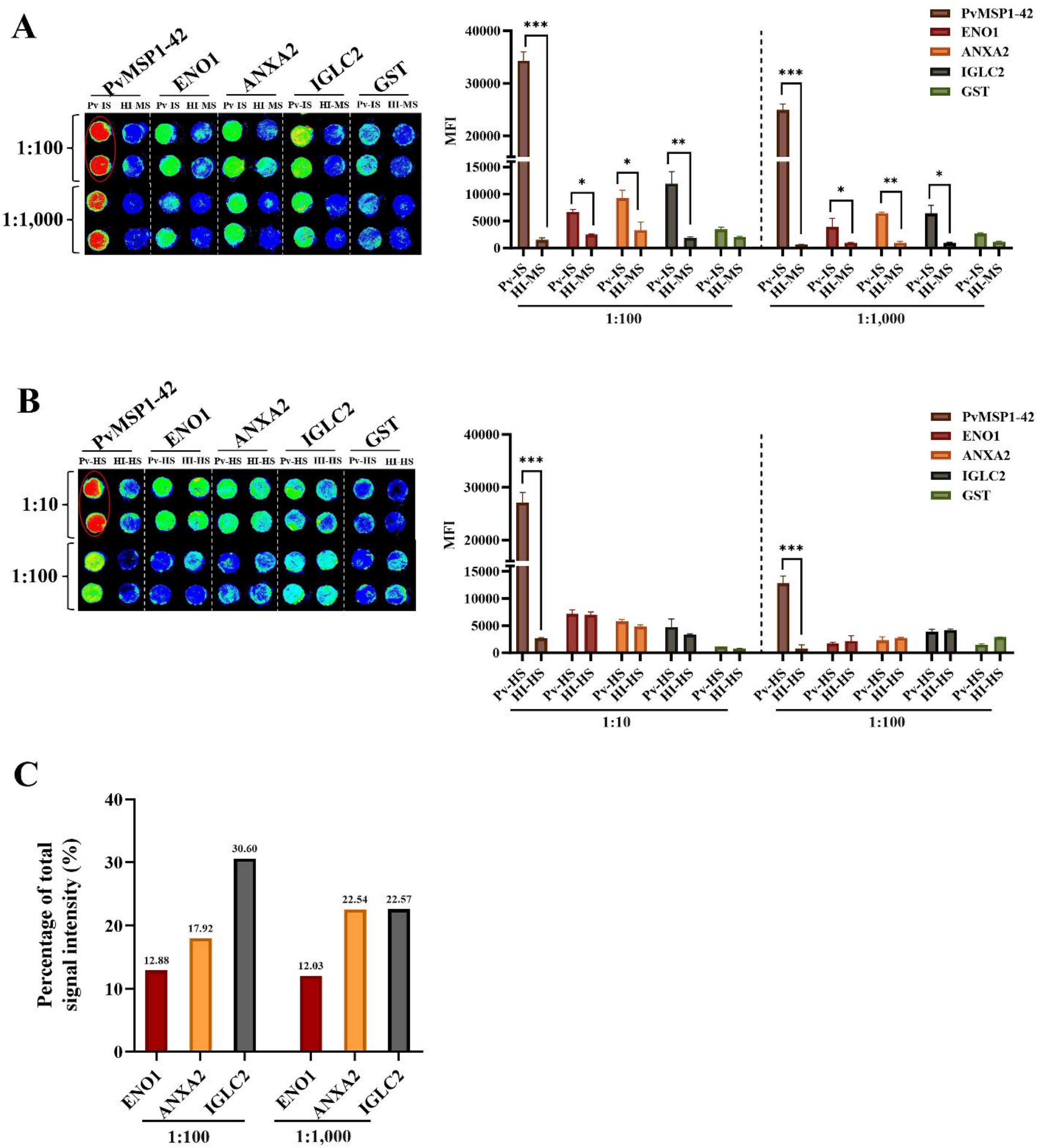
A
PvMSP1-42
ENO1
ANXA2
IGLC2
GST
Pv-IS
HI-MS
1:100
1:1,000
MFI
40000
30000
20000
15000
10000
5000
0
***
**
*
B
Pv-HS
HI-HS
1:10
1:100
C
Percentage of total signal intensity (%)
40
30
20
10
0
12.88
17.92
30.60
12.03
22.54
22.57

Fig D. Evaluation of His-tag–related background reactivity. (A) Fluorescence array images and quantification of PvMSP1-42, ENO1, ANXA2, IGLC2, and GST probed with sera from PvMSP1-42-immunized mice (Pv-IS) and healthy mice (HI-MS). **(B)** The same panel probed with *P. vivax* infected patients (Pv-HS) and healthy individuals (HI-HS). **(C)** The proportion of His-tag binding signal in the total signal intensity. Pv-HS: pooled serum from patients with acute *P. vivax* malaria; HI-HS: pooled serum from healthy human donors; Pv-IS: pooled serum from mice immunized with PvMSP1-42 protein; HI-MS: pooled serum from healthy mice.

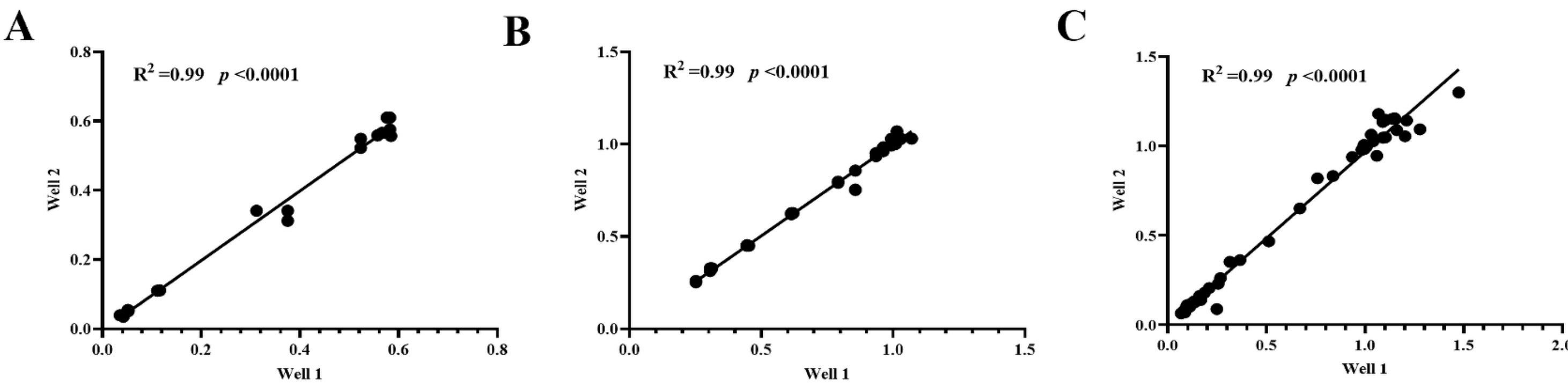

Fig E. Pearson correlation analysis of ELISA. Reproducibility was assessed by calculating Pearson correlation coefficients between optical density (OD) from duplicate wells, using serially diluted sera against PvMSP1-42.

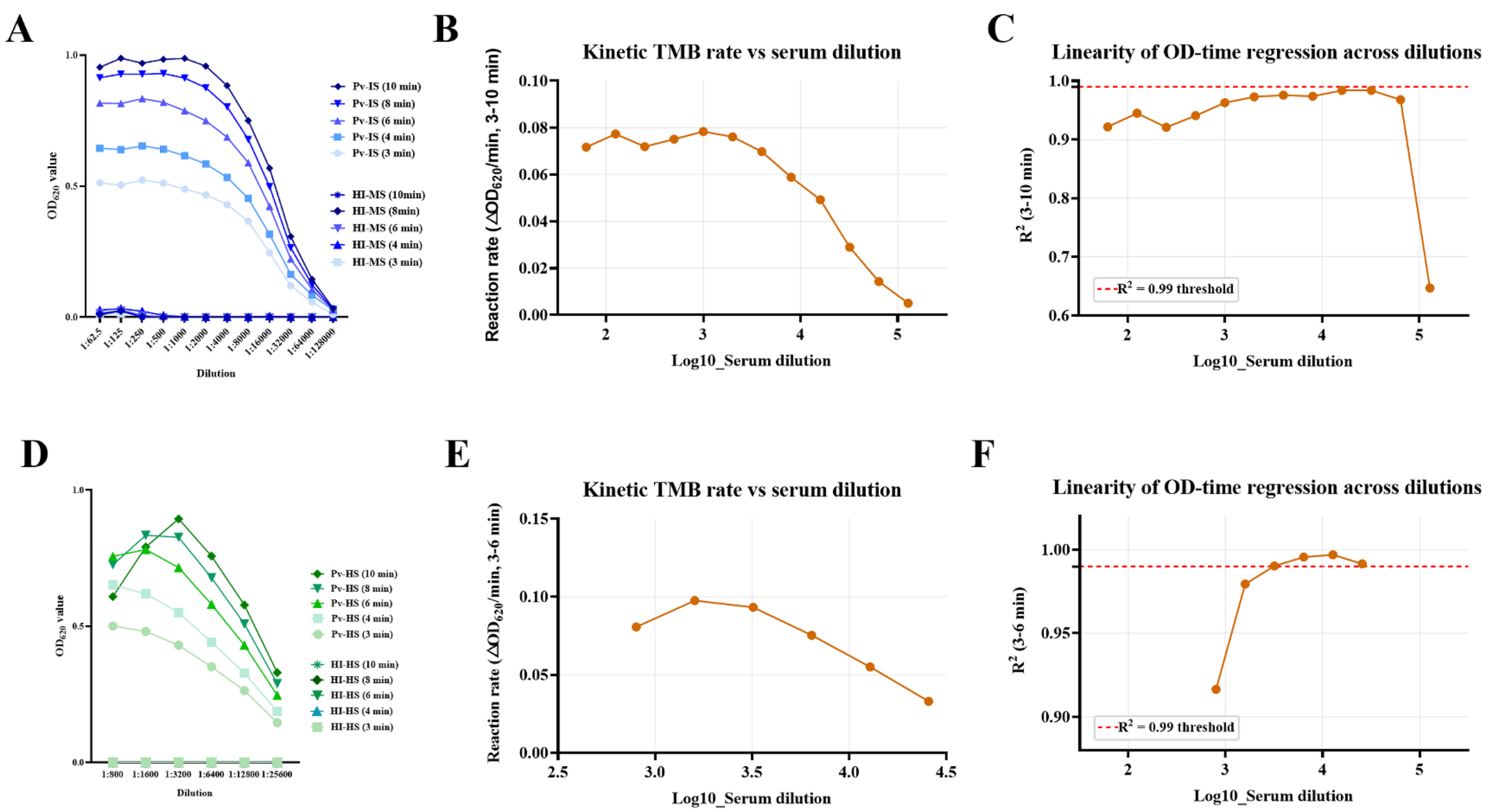

Fig F. Kinetic validation of TMB reaction and ELISA linear range. (A–C) Kinetic OD_{620} readings for serially diluted mouse sera (1:62.5–1:128,000). **(D–F)** Corresponding results for human sera (1:800–1:25,600). **(B, E)** Reaction rate ($\Delta OD/\Delta t$) across serum dilutions. **(C, F)** Regression-based R^2 analysis demonstrating the linear phase ($R^2 \geq 0.99$). Pv-IS: pooled serum from mice immunized with PvMSP1-42 protein; HI-MS: pooled serum from healthy mice; Pv-HS: pooled serum from patients with acute *P. vivax* malaria; HI-HS: pooled serum from healthy human donors.

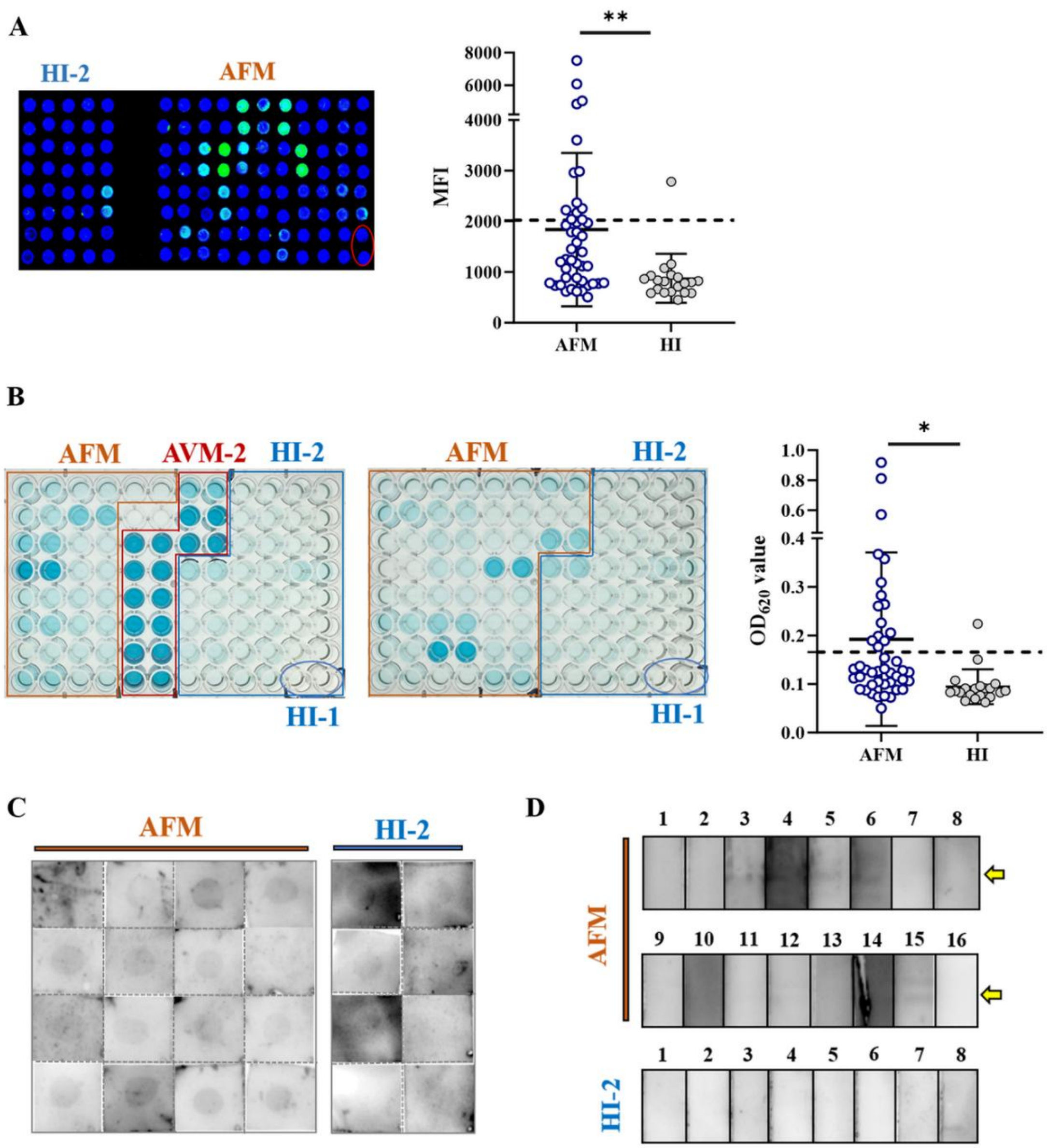

Fig G. Evaluation of potential cross-reactivity of PvMSP1-42 with sera from patients with acute *P. falciparum* malaria (AFM). (A) Detection of AFM sera by protein array using PvMSP1-42 as the target antigen. Representative array images and quantitative MFI analysis are shown. The dashed line indicates the positivity cutoff. **(B)** Detection of AFM sera by ELISA using PvMSP1-42. Representative ELISA plate images and corresponding OD_{620} values are shown. The dashed line indicates the positivity cutoff. **(C)** Representative dot blot results obtained using AFM sera and healthy control sera (HI-2). **(D)** Representative Western blot results using AFM sera (n = 16) and healthy control sera (HI-2, n = 8). Arrows indicate the expected PvMSP1-42-reactive band. Data are presented as mean ± SEM.

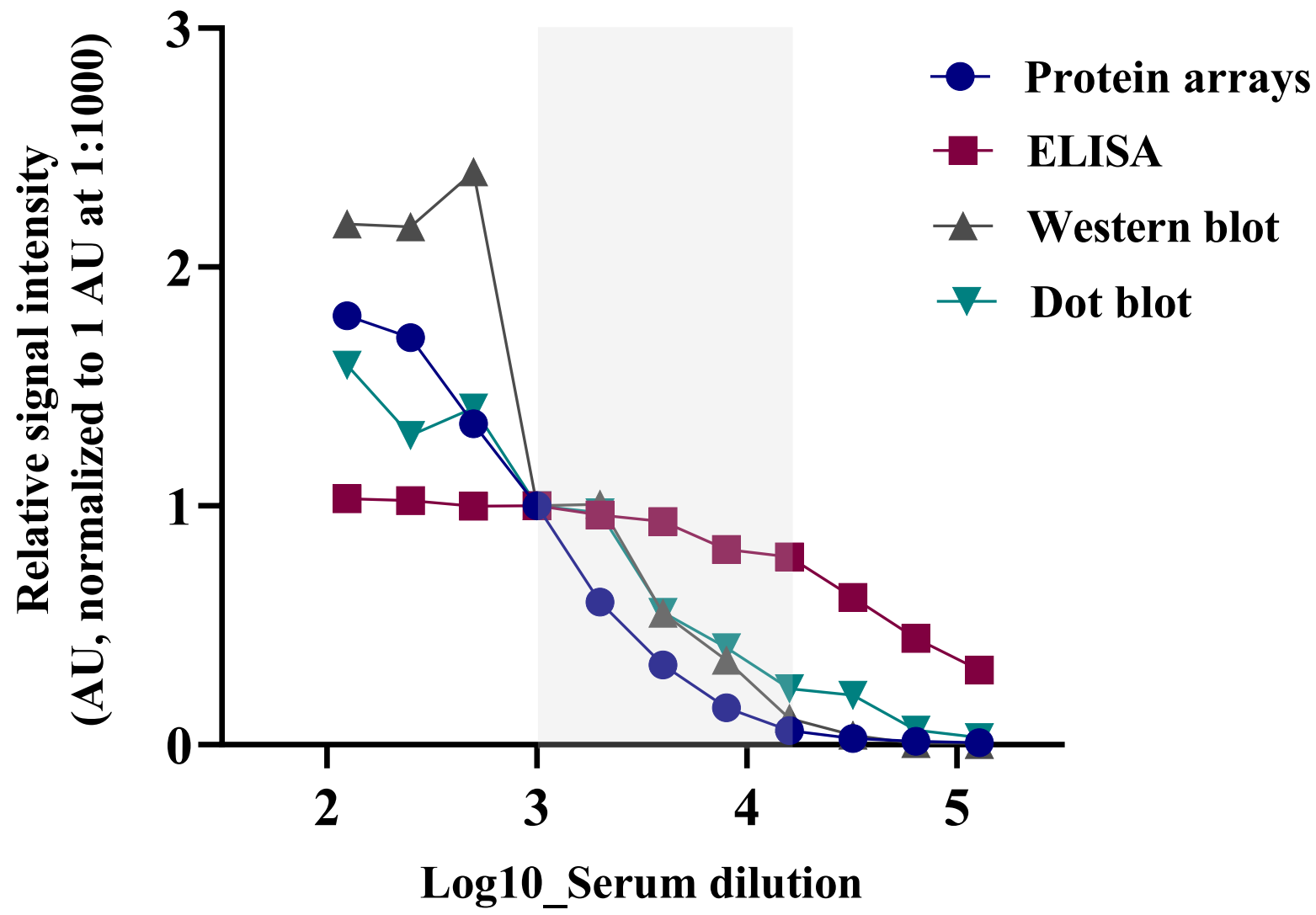

Fig H. Serial twofold dilutions of immune mouse sera (1:62.5–1:128,000) were performed across all four immunoassay platforms. Signal intensities were normalized to the value at a 1:1000 dilution, which was defined as 1 arbitrary unit (AU) and used as a shared reference calibrator to anchor signals among assays. The gray shaded area represents the overlapping linear dynamic range.

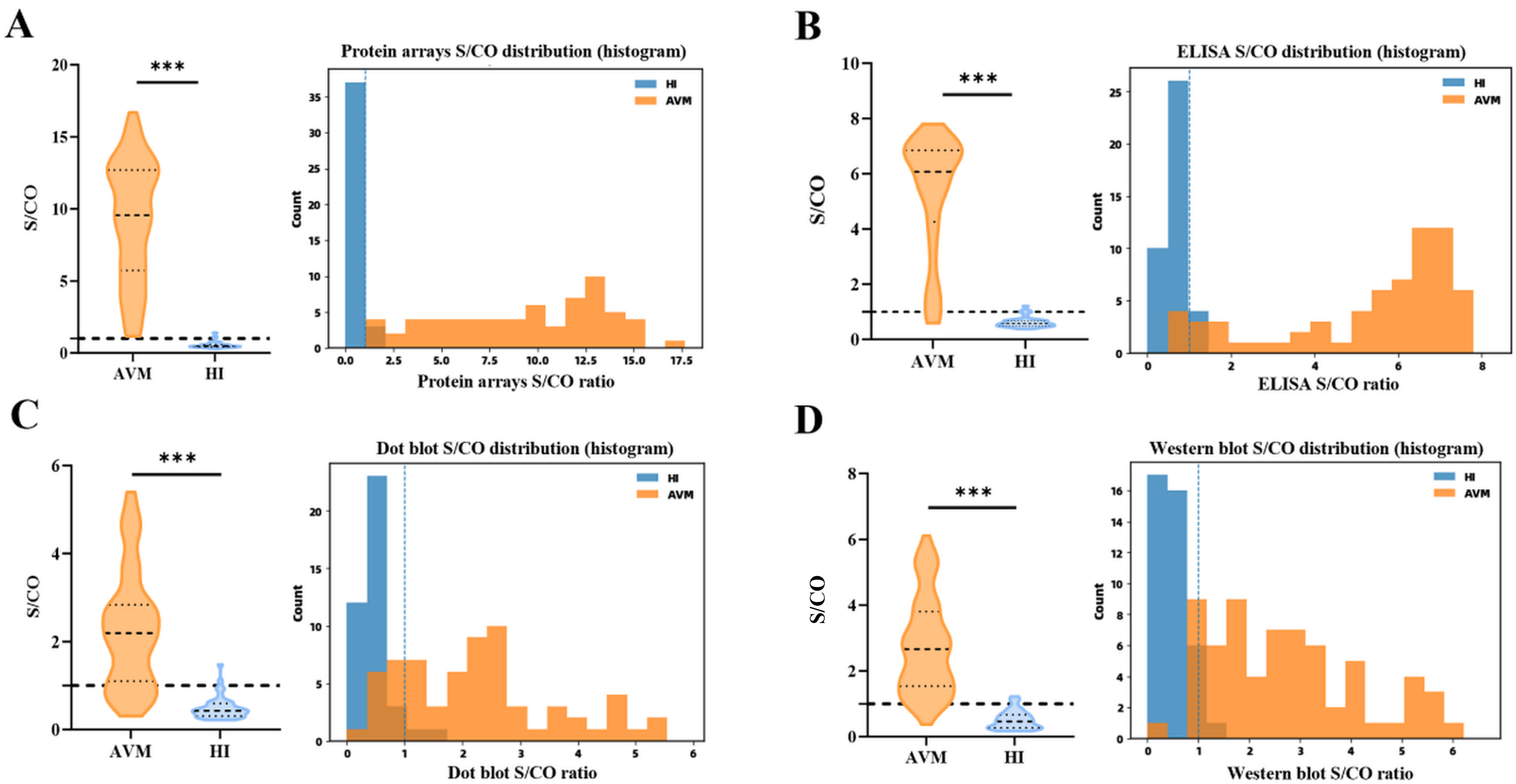

Fig I. Distribution of normalized signal-to-cutoff ratios in sera from patients with acute *P. vivax* malaria and healthy individuals across four immunoassay platforms. Violin plots and corresponding histograms show the distributions of signal-to-cutoff (S/CO) ratios obtained using **(A)** protein arrays, **(B)** ELISA, **(C)** dot blot, and **(D)** Western blot in sera from patients with acute *P. vivax* malaria (AVM, n = 66) and healthy individuals (HI, n = 40). Orange and blue indicate AVM and HI samples, respectively. The horizontal and vertical dashed lines indicate the assay-specific positivity threshold (S/CO = 1).

1.2 Supplementary Tables

Table A. Dynamic range and linearity of protein detection across three substrate-modified slides.

	Ion-modified slide		Poly-L-lysine-modified slide		APES-modified slide	
	PBS	MSD	PBS	MSD	PBS	MSD
Highest MFI	15,971 ± 4,911	21,447 ± 1,853	14,330 ± 27	19,941 ± 1,606	16,878 ± 442	7,853 ± 105
Lowest MFI	8,732 ± 1,587	6,105 ± 307	4,530 ± 299	6,040 ± 1,070	4,808 ± 326	2,645 ± 154
Blanks	4,834 ± 0.70	2,213 ± 295	2,329 ± 73	2,394 ± 385	3,080 ± 477	1,849 ± 120
Dynamic range[1]	1.83	3.51	3.16	3.30	3.51	2.96

Values are means ± SD

[1]The ratio of the highest value to the lowest.

Table B. Dynamic range and linearity of serum antibody detection across different substrate-modified slides.

	Ion-modified slide		Poly-L-lysine-modified slide		APES-modified slide	
	PBS	MSD	PBS	MSD	PBS	MSD
Highest MFI	65,442 ± 1.41	64,904 ± 648	64,904 ± 648	65,403 ± 0.70	64,571 ± 1,178	64,606 ± 1,069
Lowest MFI	2,330 ± 30	795 ± 28	2,291 ± 171	614 ± 94	1,775 ± 69	1,043 ± 123
Blanks	798 ± 268	48 ± 11	818 ± 332	87 ± 0.70	735 ± 150	28 ± 10
Dynamic range[1]	28.07	81.64	28.27	106.34	36.37	61.91

Values are means ± SD

[1]The ratio of the highest value to the lowest.

Table C. Dynamic range and detection limits of dot blot assays for proteins and serum samples.

	Protein concentration		Sera dilution	
	PBS	MSD	PBS	MSD
**Highest IntDen**	216,058 ± 45,461	185,593 ± 30,597	219,754 ± 60,657	220,063 ± 13,606
**Lowest IntDen**	7,256 ± 548	6,178 ± 125	26,993 ± 10,437	18,587 ± 233
**Blanks**	4,466 ± 1,422	2,826 ± 1,190	1,997 ±1,018	5,357 ± 700
**Dynamic range**[1]	29.77	30.04	8.14	11.84

Values are means ± SD

[1]The ratio of the highest value to the lowest.

Table D. Cross-reactivity of PvMSP1-42 with sera from patients with acute *P. falciparum* infection assessed by protein arrays and ELISA.

	Sample	No. positive	No. negative	Positive reactivity rate (%) [1]	Signal Intensity	
					MV±SEM	Highest<br>Lowest
**Protein arrays**	AFM	16	28	36	1,837 ± 227	7,526<br>504
	HI	1	19	5	876 ± 108	2,785<br>450
**ELISA**	AFM	15	29	34	0.19 ± 0.03	0.90<br>0.05
	HI	1	19	5	0.09 ± 0.01	0.22<br>0.06

[1] Positive reactivity rate = Number of sera exceeding the assay-specific positivity cutoff / Total number of sera tested × 100%.

Abbreviations: MV, mean value; SEM, standard error of measurement; AFM, acute *P. falciparum* infection; HI, healthy individuals.
